# Knowledge and practices surrounding outbreaks and COVID-19 among community health workers in rural Rwanda: a cross-sectional mixed-methods study

**DOI:** 10.11604/pamj.2023.45.35.37020

**Published:** 2023-05-15

**Authors:** Anne Niyigena, Naome Nyirahabimana, Vincent Cubaka, Vestine Mukandayisenga, Elias Ngizwenayo, Pierre Celestin Niyigena, Dale Barnhart

**Affiliations:** 1 Partners in Health, Inshuti Mu Buzima, PO Box 3432, KG9 Avenue 46, Remera, Kigali, Rwanda,; 2 Harvard Medical School, Boston, United States of America

**Keywords:** Community Health Workers (CHWs), knowledge and practice, COVID-19, Rwanda

## Abstract

**Introduction:**

Community Health Workers (CHW) are a critical resource for outbreak preparedness and response. However, CHWs´ ability to respond to outbreaks depends on their accurate knowledge of the disease and proper adoption of disease prevention practices. We explored knowledge and practices related to outbreaks in general, and COVID-19 among CHWs in Rwanda.

**Methods:**

this cross-sectional multimethod study used stratified simple random sampling to recruit three cadres of CHWs (agents de santé maternelle, female Binomes, and male Binomes) from three rural Rwandan districts. We used telephone-based data collection to administer quantitative surveys (N=292) and qualitative interviews (N=24) in September 2020. We calculated descriptive statistics and conducted thematic analysis of qualitative data. We assessed for associations between general outbreak-related knowledge and receipt of training using Chi-square tests and between COVID-19 related knowledge and CHW characteristics and adoption of prevention methods using linear regression models.

**Results:**

only 56.2% of CHWs had received training on any health topic in 12 months prior to COVID-19 pandemic and only 19.2% had specifically received training on outbreak preparedness. Almost all CHWs reported preventing COVID-19 by wearing facemasks (98%), washing hands (95%), and social distancing in crowds (89%) with fewer reporting staying at home (50%), sneezing or coughing into an elbow (38%) or using hand sanitizer (18%). Almost all CHWs in our study knew that COVID-19 transmits through respiratory droplets (98%) and by infected surfaces (98%) and that asymptomatic spread is possible (91%). However, fewer than half of community health workers correctly affirmed that children were at low risk of becoming severely ill (48%) and only 32% correctly rejected the misconception that everyone with COVID-19 would become severely ill. There was no association between COVID-19-related knowledge and adoption of COVID-19 preventative practices. Qualitative findings suggested that while CHWs possessed lots of correct information about COVID-19 and reported good adherence to COVID-19 prevention practices, they also commonly held misconceptions that over-exaggerated the dangers of COVID-19.

**Conclusion:**

gaps in knowledge, training, and access to information point to a need for additional investment in supervision and credible informational systems to support CHWs.

## Introduction

Community health workers (CHW) are key personnel in health systems around the world and support a wide range of health services, including malaria control, family planning, maternal and child health, infectious disease, and other decentralized health programs [1,2]. In addition to providing routine health services in the communities, CHWs are increasingly incorporated into national outbreak preparedness planning [3]. Early in the COVID-19 pandemic, the international community recognized that CHWs´ trusted position in the community, training and experience in disease prevention and management would allow them to play critical roles in the COVID-19 response, including community education, contact tracing, and maintaining routine health service delivery [4-7]. The recognition of the role of CHWs in COVID-19 response was in part grounded in the notion of adaptability of past lessons from health crisis to optimize COVID-19 response [5,8-10]. In the context of other outbreaks, health workers who had received general emergency training reported higher readiness to respond to tuberculosis outbreak in Nigeria and Ebola virus in Saudi Arabia and Ghana [11-13]. Similarly, research has shown that healthcare workers with prior outbreak experience were more likely to translate their tacit knowledge and perceptions to COVID-19 when robust information about the virus was yet to be available, and hence were able to cope with pandemic anxiety while responding to COVID-19 pandemic [14-16]. Nevertheless, when CHWs were first engaged in COVID-19 response activities, little was known about their COVID-related knowledge, attitudes, and practices regarding COVID-19. Research conducted in Ethiopia, India, Nigeria and Mozambique early in the pandemic suggests that CHW knowledge about COVID-19 was suboptimal, with mixed evidence on the extent to which CHWs adhered to preventative practices [17-20]. However, these studies also indicate that knowledge levels and adherence to prevention practices can vary substantially by time, location, CHW´s educational background, and the specific dimensions of COVID-19 knowledge that are assessed. For example, Feldman *et al*. 2021 reported that while CHWs in their study described good overall knowledge of COVID-19, they were less likely to correctly identify high-risk groups (2%) and transmission routes (16%) than to identify correct symptoms (25%) and prevention measures of COVID-19 (39%) [18]. Similarly, CHWs from settings with higher levels of education and those struck by outbreaks before reported higher level of COVID-19 knowledge early in the COVID-19 pandemic [21-23]. Rwanda registered its first COVID-19 case on 14^th^ March 2020 and reported 20,975 cases and 295 deaths in the first year of the pandemic [24]. Since the start of the pandemic, Rwandan CHWs have played a key role in the provision of community health care services at the community level and helped to support the national COVID-19 response in several ways including contact tracing and linkage to testing as well as community awareness [25,26]. Although previous research has reported good COVID-19 knowledge and adherence to preventative practices among facility-based health care workers in Rwanda [27], knowledge and practices among Rwandan CHWs have not been previously investigated. This study explored general outbreak-related knowledge as well as COVID-specific knowledge among CHWs from three districts in rural Rwanda.

## Methods

**Study setting:** this study occurred in three rural Rwandan districts: Burera, which is in the Northern province, and Kayonza and Kirehe, which are in the Eastern province. These districts are supported by Partners In Health/Inshuti Mu Buzima (PIH/IMB), an international organization that has been operating in Rwanda since 2005 and supports the Ministry of Health through health system strengthening and providing health care to vulnerable populations. In these three districts, as elsewhere in Rwanda, community-level health care services are delivered by CHWs. Each Rwandan village of approximately 100 to 250 households has three CHWs: one female *Agent de Santé Maternelle* (ASM) provides child and women´s health services, and a male-female pair of *Binomes* care for childhood illness, malaria, family planning services, and other diseases. CHWs are elected by their village neighbors and are required to know how to read and write, be 20-50 years old, and have a reputation for trustworthiness and integrity. Once elected, they receive training on health care provision by the Ministry of Health. CHWs serve as volunteers , but received a small incentive based on their performance around key health indicators, such as TB, HIV and stunting [28]. While Rwanda doesn´t have alarming history of outbreaks, Rwanda is surrounded by countries that have had devastating outbreaks such as Ebola, cholera, meningitis and poliomyelitis. However, when an outbreak emerges in neighboring countries, Rwanda activates prevention and surveillance efforts that usually start in the communities and are implemented by CHWs. Moreover, all CHWs in Rwanda are provided with a kit that contains essential supplies they need in the delivery of health services in the community. They also receive a cell phone by the virtue of their duties, which they use for communication and for reporting.

**Study design and population:** this study used a cross-sectional multimethod approach to learn about outbreak and COVID-19-related knowledge and practices among CHWs in Rwanda. We used a stratified simple random sampling method to select quantitative survey respondents from the 5,769 CHWs serving across the three PIH/IMB-supported districts. CHWs were cross-classified by districts (Kirehe, Burera, Kayonza) and cadres (ASM, female Binome, male Binome), which made 9 strata to sample from. Due to logistical and time considerations associated with phone-based data collection, we estimated to sample 5% of CHWs from each stratum which resulted in a targeted sample size of 292. This sample would allow us to report 95% confidence intervals with a precision of at least ±6% for the overall CHW population, district-specific estimates with a precision of at least ±14%, and cadre-specific estimates with a precision of at least±12%. The 5% of CHWs in each stratum was randomly selected from CHWs sampling frame and those who were unreachable on the phone were replaced by the next CHWs on the list, which expanded the overall sample size to 349. We excluded CHWs who had been serving their communities for less than a year from data collection. Full details on the process of our sampling and participants´ flowchart is available elsewhere [26].Qualitative interview participants were purposively selected from CHWs who were not quantitative survey respondents.

**Data collection:** we administered a quantitative survey via telephone in September 2020, which was 6 months since the start of the COVID-19 pandemic in Rwanda. Survey questions around trainings in general, availability of supplies and background information were adapted from an existing questionnaire previously used to evaluate the Rwanda MOH´s Community Performance-Based Financing program [18], while questions around COVID-19 knowledge and prevention practices were developed based on the literature [29-31]. Data were collected in REDCap using tablets and directly uploaded to the REDCap server hosted by PIH/IMB [21]. Qualitative data were collected using a pre-designed interview guide. Qualitative interviews were also administered through phone calls and were audio recorded. All data collection was conducted in Kinyarwanda by trained data collectors. During the qualitative interviews, we asked open-ended questions also adapted from questions that similar studies from similar settings have inquired The questions mainly explored what CHWs know about COVID-19 in general, about how it is spread, ways of containing its spread, the level of risk and general knowledge about outbreaks.

**Data analysis:** we described the demographic characteristics of participants including district, CHW cadre; gender; age; education; and socio-economic status measured using the Rwandan government´s ubudehe system, where category 1 reflects acute poverty and category 3 reflects households with surplus income, using frequencies and proportions for categorical variables and medians and interquartile ranges (IQRs) for continuous data. We also reported frequencies and statistics related to general outbreak preparedness, including whether or not the CHW reported having received any training during 12 months prior COVID-19 pandemic, whether or not they had received training on outbreak preparedness during 12 months prior COVID-19 pandemic, ownership of and ability to use thermometers, and, among Binomes, ownership and ability to use rapid diagnostic tests (RDTs). We assessed CHW´s sources of COVID-19 knowledge, engagement with the national COVID-19 response, and practice of COVID-19 prevention activities using a series of open-ended questions where data collectors were trained to check responses from a pre-defined list of responses. The practice of COVID-19 prevention activities was inquired using an open-ended question “how do you protect yourself from COVID-19?” To understand CHWs general outbreak-related knowledge we asked CHWs the open-ended question, “What do you understand when a word “outbreak” is mentioned?” Data collectors conducted real-time coding of responses using a pre-defined list that included an outbreak attacks many people in a very short time, an outbreak is contagious, an outbreak can have a zoonotic origin or be caused by viruses, contact of cases should be identified and screened and suspected cases should be reported to the health center. Data collectors were also encouraged to use an “other” option to capture unanticipated knowledge. This data was analyzed by first reviewing the free-text responses and then either included them into one of the existing pre-defined categories or generating a new category that reflected the response. We then reported the percentage of respondents mentioning each reason.

COVID-19-related knowledge was assessed using a series of 12 True-False questions. Eleven statements were taken from a prior survey in Malawi [31], and a twelfth question, “Black skinned people are at lower risk of COVID-19” was added to the list by our staff based on a common rumor circulating at the time of the survey. For each item, participants could either agree, disagree, or say they did not know. We reported the proportion of CHWs providing a correct response to each of the twelve items. A correct response for each knowledge item was given a score of one, and with twelve knowledge questions, scores of numbers of correct responses ranged between 0 and 12. The mean scores of correct responses to COVID-specific questions and the corresponding 95% confidence intervals were reported for the overall study population and by CHWs characteristics. We also used linear regression to assess the association between the mean scores of correct responses and CHWs´ demographic characteristics, engagement in national COVID-19 response, and use of prevention methods. The level of significance was set at p-value <0.05 Qualitative data was transcribed and translated from Kinyarwanda to English. A codebook was developed using an inductive thematic analysis. This codebook was used to conduct a deductive analysis and identify final themes. All quantitative analysis was performed using Stata v15.0 [21] and qualitative analysis was performed using MAXQDA software.

**Ethical approvals and informed consent:** this study received ethical clearances from the Rwanda National Ethics Committee (881/RNEC/2020) and the Partners In Health/Inshuti mu Buzima Research Committee. Since the study was conducted during COVID-19 lockdown, the Rwanda National Ethics Committee had recommended phone-based data collection to prevent physical interactions that may put research staff or the communities at risks for COVID-19 infection. We received verbal consent with a confirmatory text message from all participants. Personal identity including phone numbers were kept in a safe storage different from the data storage and that information were destroyed at the completion of data validation.

## Results

**Figure 1:**
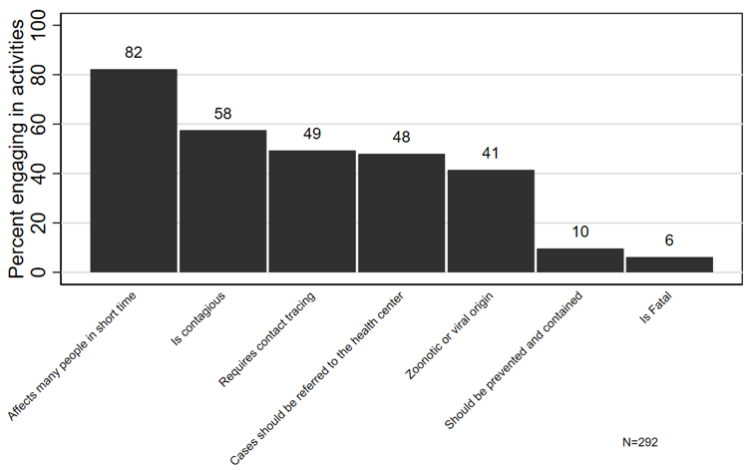
general knowledge about outbreaks among community health workers (N=292)

**Figure 2:**
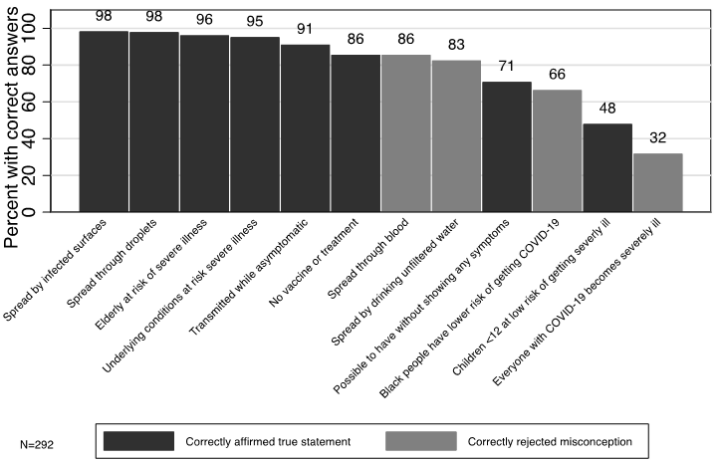
COVID-19 knowledge among community health workers (N=292)

Of 349 CHWs who were sampled, 292 (83.6%) consented to participate in the quantitative survey. Of the 292 participating CHWs, 43.1% were from Kirehe, 38.4% were from Burera, and 18.5% were from Kayonza (Table 1). Three-quarters were Binomes (75.0%), most were female (63%), and over 90% had completed primary school. Only 56.2% of CHWs had received training on any health topic in 12 months prior the pandemic and only 19.2% had specifically received training on outbreak preparedness in 12 months prior COVID-19 pandemic. Although 70.2% of CHWs owned a thermometer and were very confident in using it, 13.0% of CHWs did not own thermometers at all. Similarly, while 84.5% of Binomes reported that they had RDTs for malaria and were very confident using them, 5.9% never used them *at al*. The most common sources of COVID-19 knowledge were mass media (99.7%) followed by local government leaders (56.2%, Table 2). Only 1.7% of CHWs reported trainings as a primary source of COVID-19 knowledge. Nearly all CHWs (98.3%) were engaged in at least one COVID-19 response activity, with almost all CHWs supporting community education and mobilization (95%). Almost all CHWs wore facemasks (98%) and washed their hands (95%) and the majority reported social distancing in crowds (89%). Fewer reported staying at home (50%), sneezing or coughing into an elbow (38%) or using hand sanitizer (18%). When asked to free-list their general knowledge about outbreaks, 82.2% reported that an outbreak attacks many people in a very short time, slightly over half (57.5%) knew that outbreaks were contagious, less than half mentioned that contact of cases should be identified and screened (49%) or that suspected cases should be reported to the health center (48%) or that outbreaks could have a viral or zoonotic origin (41%). A few CHWs mentioned that outbreaks should be prevented and contained (10%) or that an outbreak is fatal (6%, Figure 1). On average, CHWs correctly answered 9.5 out of 12 questions about COVID-19 (95% CI: 9.3, 9.7). Over 95% of CHWs knew that infected surfaces and respiratory droplets were possible routes of COVID-19 transmission and that the elderly and those with chronic conditions were at higher risk from COVID-19 (Figure 2). Conflictingly, although 91% knew that COVID-19 could be transmitted while asymptomatic, only 71% knew it was possible to have COVID-19 with no symptoms. Most CHW also correctly affirmed that there was no vaccine or treatment for COVID-19 at the time of data collection. Most also rejected drinking water and blood as modes of transmission and rejected that black people were at low risk of COVID-19. However, fewer than half of CHWs correctly affirmed that children under 12 were at low risk of becoming severely ill and only 32% correctly rejected the misconception that everyone with COVID-19 would become severely ill. Neither demographic characteristics nor receipt of training during 12 months prior the pandemic were associated with the number of correct COVID-19 responses (Table 3). Citing educational campaigns as a source of COVID-19 knowledge was associated with worse COVID-19 knowledge (9.2 vs 9.7, p=0.002). Community health workers correctly responded to slightly more facts about COVID-19 if they participated in the national COVID-19 response by providing community education (9.5 vs 8.7, p=0.029) or re-integration of recovered COVID-19 patients (10.6 vs 9.5, p=0.036). Knowledge was not associated with engagement in any COVID-19 prevention practices. Qualitative findings: twenty-four CHWs participated in qualitative interviews, including 15 females and 9 males CHWs. CHW´s knowledge was classified in four themes a) perceptions of COVID-19 severity b) knowledge about groups who were at high risk of COVID-19, c) knowledge about COVID-19 transmission, and d) knowledge about prevention measures. We also compared CHWs knowledge by CHW cadre and the district of residence. Perceptions of COVID-19 severity: CHWs universally perceived COVID-19 to be a serious health threat, with many emphasizing that it could be deadly. They generally also knew that it is preventable, even if no vaccine or treatment existed. “COVID-19 is a pandemic which spread across the globe and has no vaccine so far. Until now, there is no cure nor vaccine; other is that it is possible to prevent it” A Female Binome CHW.

**Table 1:** background characteristics of study participants (N=292)

Variables	Number (N)	Percentage (%)
**District of residence**		
Burera	112	38.4
Kayonza	54	18.5
Kirehe	126	43.1
**CHW type**		
ASM	72	24.7
Binome	220	75.3
**Gender**		
Female	194	66.4
Male	98	33.6
**Age1**		
≤39	93	32.5
40-49	120	42.0
≥50	73	25.5
**Level of education**		
Incomplete primary (<6years of basic education)	26	8.9
Completed primary school (6 years basic education)	173	59.3
Enrolled in or completed secondary school (<=6 years of high school)	93	31.8
**Ubudehe category2**		
Ubudehe 1	22	7.6
Ubudehe 2	126	43.3
Ubudehe 3	143	49.1
**Receivedany training 12 months prior the pandemic**	164	56.2%
**Received training on outbreak preparedness 12 months prior COVID-19 pandemic**	56	19.2%
**Thermometer ownership and confidence**		
Very confident	205	70.2%
Confident	39	13.4%
Less confident	8	2.7%
Not confident	2	0.7%
No thermometer	38	13.0%
**RDT usage and confidence (N=220)3**		
Very confident	186	(84.6%)
Confident	21	(9.6%)
Less Confident	1	(0.5%)
Never used an RDT	12	(5.5%)

1 Age was missing for six individuals. ^2^Ubudehe category was missing for one individual. Assessed among Binomes only.

**Table 2:** engagement with sources of COVID-19 knowledge, the national COVID-19 response, and COVID-19 prevention activities (N=292)

Variables	Number (N)	Percentage (%)
**Sources of COVID-19 knowledge1**		
Mass media (TV, Radio, newspaper)	291	99.7
Local government leaders	164	56.2
Friends or relative	110	37.7
Educational/information campaigns	102	34.9
Training	5	1.7
**Role in national COVID-19 response1**		
Educating the community about COVID-19	278	95.2
Filling gaps in service provision	150	51.4
Referring suspected cases to the health facility	62	21.2
Case finding	24	8.2
Availing resources in the community	18	6.2
Contact tracing	11	3.8
Reintegrating recovered COVID-19 patients	7	2.4
Not supporting the COVID-19 response effort	5	1.7
**COVID-19 prevention practices1**		
Wear face mask	287	98.3%
Handwashing with soap and water	276	94.5%
Stay ≥1 meters from others in a crowd	259	88.7%
Stay at home	145	49.7%
Cough and sneeze into elbow	110	37.7%
Use hand sanitizer	54	18.5%

1 CHWs could cite multiple sources of COVID-19 knowledge, multiple roles in the national COVID-19 response effort, and multiple COVID-19 prevention practices.

**Table 3:** average number of correct responses to 12 COVID-19 knowledge questions by demographic characteristics, engagement in national COVID-19 response, and use of prevention methods (N=292)

Variables	Number of correct responses (Mean, 95% CI)			P-values
**District Overall mean score of correct**	**Burera**	**Kayonza**	**Kirehe**	
**responses**	9.4 (9.1, 9.6)	9.7(9.3, 10.2)		0.320
**CHW type**	**ASM**	**Binome**		
	9.4 (9.1, 9.7)	9.5 (9.3, 9.7)		0.506
**Gender**	**Male**	**Female**		
	9.6 (9.4, 9.9)	9.4 (9.2, 9.6)	9.5 (9.3, 9.8)	0.197
**Age2**	**≤39**	**40-49**	**≥50**	
	9.5(9.2, 9.8)	9.5 (9.3, 9.8)	9.5 (9.1, 9.8)	0.925
**Level of education**	**Incomplete primary**	**Completed primary**	**Beyond primary**	
	8.9 (8.4-9.5)	9.5 (9.3, 9.7)	9.6 (9.3, 9.9)	0.073
**Ubudehe category^3^**	**1**	**2**	**3**	
	9.5 (8.9, 10.1)	9.5 (9.2, 9.7)		0.859
**Any training 12 months prior the pandemic**	**Yes**	**No**		
	9.6 (9.4, 9.8)	9.3 (9.1, 9.6)		0.078
**Training on outbreak**	**Yes**	**No**		
**preparedness 12 months prior the pandemic**	9.6 (9.2, 10.0)	9.5 (9.3, 9.6)		0.505
**COVID-19 knowledge source**	**Yes**	**No**		
Mass media	9.5 (9.3, 9.6)	12.0 (9.3, ---4)		0.068
Local government leaders	9.4 (9.2, 9.6)	9.6 (9.3, 9.8)		0.372
Friends/relatives	9.5 (9.2, 9.7)	9.5(9.3, 9.7)		0.818
Information campaigns	9.2 (8.9, 9.4)	9.7 (9.5, 9.9)		0.002
Training	9.6 (8.4, 10.8)	9.5 (9.3, 9.7)		0.866
**Participation in national COVID-19 response**	**Supported**	**Did Not Support**		
Educating the community	9.5 (9.4, 9.7)	8.7 (8.0, 9.4)		0.029
Filling gaps	9.6 (9.4, 9.8)	9.4(9.2, 9.6)		0.328
Referring suspected cases	9.8 (9.4, 10.1)	9.4 (9.2, 9.6)		0.092
Case finding	9.4 (8.9, 10.0)	9.5 (9.3, 9.7)		0.767
Availing resources	9.8 (9.2, 10.5)	9.5 (9.3, 9.6)		0.285
Contact tracing	9.5(8.7, 10.4)	9.5(9.3, 9.7)		0.905
Reintegrating recovered patients	10.6 (9.6, 11.6)	9.5 (9.3, 9.6)	9.5 (9.3, 9.8)	0.036
**COVID-19 prevention practices1**				
Face mask	9.5 (9.3, 9.7)	9.0 (7.8, 10.2)		0.417
Handwashing	9.1(8.4, 9.7)	9.5 (9.4, 9.7)		0.195
Stay ≥1 meter from others	9.5 (9.3, 9.6)	9.7(9.2, 10.2)		0.375
Stay at home	9.4(9.2, 9.6)	9.6 (9.4, 9.8)		0.202
Cough and sneeze into elbow	9.6(9.4, 9.9)	9.4 (9.2, 9.6)		0.278
Use hand sanitizer	9.6 (9.3, 10.0)	9.5 (9.3, 9.6)		0.432

P-value from a chi-squared test from a linear regression model containing the explanatory variable to an intercept only model. 2N=286. 3N=291. 4Upper bound of the 95% CI exceeded maximum number of questions on the questionnaire.

However, in some cases the CHW appeared to overestimate the chances of death from COVID-19. *“What I know on this pandemic is that it kills whoever does not protect against it. If you don´t wash your hands, put on facial masks and respect social distancing.”* A male Binome CHW.

CHWs also portrayed COVID-19 as the most contagious and fatal disease compared to other diseases they have known. *“Covid-19 is a highly contagious disease that is different from all other diseases because it infects in a short period and kills”* A Male Binome CHW.

**Knowledge about groups at high risk of COVID-19:** many CHWs recognized that all people could be infected with COVID-19. Many also correctly knew the elderly, pregnant women, and people with previous conditions were at higher risk of severe complications from COVID-19. *“…. those who are more vulnerable are those whose body immunity is low, like people aged above 50, under-5 children, pregnant women, those people who have chronic diseases like diabetes, cancer, and high blood pressure, … everyone can contract coronavirus but mostly those groups are more vulnerable.”* A female ASM CHW

More CHWs understood the risks of COVID-19 from the immunity perspective and tended to mistakenly include children among groups at high risk “The highest risk of COVID-19 infection are people over the age of 65 years… Even those who are under five years of age, they are still weak and have less immunity and body protection”. A female Binome CHW Some strongly emphasized that COVID-19 could infect anyone, but did not seem to recognize that risks were different for different groups. “What I do know about this epidemic, is that it infects anyone, and can kill anyone” A male Binome CHW.

**Knowledge about COVID-19 transmission:** many CHWs knew that COVID-19 is contagious and correctly identified routes of COVID-19 transmission: *“It is transmitted through physical contact with liquid particles from an infected person, such as saliva and liquid particles released when someone breathes, and by touching a surface where an infected person has touched or left droplets from the mouth or nose.”* A male Binome CHW.

Some of them mentioned additional routes of transmission that were incorrect, such as transmission through sweat or semen or fecal-oral routes. CHWs who identified other incorrect routes were often referring to the importance of hand hygiene *“You know the liquid that is in people´s hands, when people have touched on an object multiple times and that someone else touches the same object and that maybe he/she has the disease, it is also another way that it can be spread; mostly that is the way it spreads”*. A Male Binome CHW.

However, other CHWs who indicated misconception around routes of transmission suggested COVID-19 as a deadly disease that cannot be avoided even if one is wearing a protection. *“It is especially contracted through body fluids such as sweat, nasal mucus and semen. Any contact with the virus from such fluids will lead to inevitable contamination regardless of your clothing or of who you are”* A Female ASM CHW.

**Knowledge about prevention measures:** CHWs had a good knowledge of COVID-19 prevention measures. Most correctly listed practices like hand hygiene, social distancing, the use of face mask, and good coughing practices. They emphasized that awareness campaign was the key to communicate these prevention practices. *“Prevention measures include regular hand washing with clean water and soap. On top of that, we have to wear face masks and keep the practice of physical distancing, at least one meter apart whenever we are in a crowded area.”* A male Binome CHW.

While hand hygiene was perceived as key prevention measure, two CHWs also articulated hand hygiene in the context of preventing oral-fecal transmission *What we can do to prevent COVID-19 is to avoid to touch any toilet doors we don´t know anyone who touched on it, and when we get out of the toilet, wash our hands, with clean water and soap.”* A female Binome CHW.

One CHWs also emphasized the importance of collective effort in preventing against COVID-19 *“The most important prevention measure is to sensitize people - when there is a community meeting in the village-about coronavirus and educating them on the signs and symptoms of the virus, and showing them the materials to use and tell them that they must buy that protective equipment in case they don´t have them, in order to stay protected.”* A female ASM CHW.

No COVID-19 vaccines were available at the time of data collection, and only one CHW mentioned the perceived importance of vaccines in the future. *“Unless a vaccine is found and we get vaccinated, otherwise we will all get infected.”* A female ASM CHW.

**Knowledge of COVID-19 by gender, CHW cadres and district of residence:** Sixteen (16) out of 24 CHWs included in this qualitative exploration exhibited at least one incorrect or exaggerated understanding about COVID-19, and female (12 out of 15) were more likely to have misconceptions than male (4 out of 9). All male who identified incorrect information tended to view pregnant women as a group at higher risks while the majority of female viewed children as a most vulnerable group. Among female, incorrect knowledge around transmission and preventions tended to implicate body fluids, including sweat as well as limited body hygiene as the primary route of transmission of COVID-19. Furthermore, CHWs whose duties is to cater for a wide range of diseases (*Binome* CHWs) made the majority (7 out 8) of CHWs who did not share incorrect information around COVID-19. CHWs who reside in district that had the highest COVID-19 infection rate (4 out 6) were more likely to articulate correct knowledge of COVID-19.

## Discussion

Our study assessed knowledge, attitudes, and practices surrounding outbreaks and COVID-19 among Rwanda´s Community Health Workers during the first 6 months of the COVID-19 pandemic in Rwanda. Both quantitative and qualitative findings indicated that CHWs in our study possessed correct information about outbreaks in general and COVID-19 in particular. Knowledge surrounding COVID-19 transmission was extremely good with almost all CHWs in our study knowing that COVID-19 transmits through respiratory droplets and by infected surfaces and that asymptomatic spread is possible. As has been previously reported among facility-based health care workers in Rwanda [27], CHW´s use of face masks, handwashing with soap and water, and keeping distance from others in a crowd was extremely high. This adherence to prevention measures was higher than what was reported among CHWs in other African countries [18,19]. However, we did not observe associations between COVID-19 knowledge and engaging in preventive activities. The high adherence to preventive practices reported in the present study might reflect strict COVID-19 control policies in Rwanda, which mandated universal adherence to COVID-19 prevention [22], suggesting policies, norms, and other contextual factors may be bigger drivers of behavior change than knowledge in this setting.

However, CHWs also reported misconceptions around populations at highest risk, routes of transmission and the severity of COVID-19. Generally, these misconceptions tended to over-exaggerate the severity or transmissibility of COVID-19, perhaps due to underlying fear and anxiety around the pandemic. Because CHWs play a major role in educating their community about COVID-19, misconceptions and fears among CHWs could be amplified throughout their communities. On the bright side, the extent to which CHWs exaggerated the danger of COVID-19 may have motivated them to mobilize their community to adhere to COVID-19 prevention practices early in the pandemic [23,28]. However, in other settings, perceptions that the dangers of the COVID-19 pandemic were over-exaggerated have been associated with reduced long-term adherence to COVID-19 prevention activities [24]. Early in the pandemic, public health communications tended to emphasize using a traditional educational strategy focused on communicating correct information about COVID-19 rather than proactively countering misinformation using “debunking” or “pre-bunking” strategies [2,25]. Neutral educational communication campaigns would not necessarily provide CHWs with sufficient information to reject misconceptions. Ensuring that CHWs have accurate information about the limitations as well as the dangers of COVID-19 and other diseases may help reduce stress among CHWs and maintain community trust in CHWs as a reliable source of information.

Our findings also point to broader gaps in the current CHWs system which should be addressed to enhance preparedness for future outbreaks. Only slightly over half of CHWs had received any training 12 months before COVID-19 pandemic and less than a fifth had be trained on outbreaks. Although CHWs also reported high confidence level in using thermometers and RDTs, not all CHWs had these essential supplies. The low levels of hand sanitizer use in this study may have similarly reflected a lack of supplies rather than a lack of engagement with the practices. In our study, receiving COVID-19 information via informational campaigns was significantly associated with worse COVID-19 knowledge, which may suggest that these campaigns were poorly designed for their target audience or, as discussed above, did not sufficiently address misinformation. Collectively, these findings point to the importance of investing in the CHW program, especially in the creation of flexible communication strategies that can quickly respond to future outbreaks [26]. For example, SMS-based communication platforms have recently emerged as a cost-effective communication model to supplement in-person training, mentorship and supervision for healthcare workers in resource-constrained settings [27,28]. In Rwanda, all CHWs receive basic cellphones, and so investing in this sort of system could allow for CHWs to receive early and frequent information about COVID-19 or other future health crises.

This study is one of the few to assess COVID-19 and general outbreaks knowledge, practice and readiness among CHWs in Rwanda and sub-Saharan Africa during early period of the pandemic and exhibited some strengths. First, its mixed methods design offered the opportunity to capture how CHWs explain their knowledge and practice around COVID-19 in their own words, and laid out the contexts in which misconception were articulated. Second, we illustrate that it was feasible to leverage existing infrastructure such as cell phones to carry out primary research activities during COVID-19 lockdown in a resource constraint setting, without putting researchers and participants at risk for infection. Thirdly, phone-based qualitative interviews were a novel aspect of this study.

However, this study also had several limitations. First, data was self-reported and vulnerable to social desirability bias, particularly as our data collection team is affiliated with a health systems non-governmental organization that regularly interacts with and provides support to CHWs. To mitigate this source of bias, our study team did not include individuals who are regularly involved with the CHW program. Second, this study was cross-sectional and was conducted early in the pandemic; therefore, it may not reflect changes in knowledge or practices over time. Third, although our survey tools were based on pre-existing surveys conducted in similar settings, these questions were adapted to the context of the COVID-19 pandemic and these changes were not validated prior to implementation. Fourth, we used open-ended questions to assess general outbreak knowledge and engagement in COVID-19 prevention activities which implies that CHWs may not be able to exhaustively report on these dimensions. Fifth, we used a parallel convergent approach where qualitative data was collected concurrently with quantitative data, therefore, we were not able to use the qualitative interviews to fully explore unexpected findings from our quantitative analysis, as would have been possible if we had chosen a sequential mixed methods design. Finally, when comparing our findings with previous research on COVID-19-related knowledge and practices among CHWs, we often discovered that the existing literature did not provide sufficient details on their knowledge and practice assessment questions to allow for a meaningful comparison of our findings to previous studies. We would encourage future researchers to provide details on the specific phrasing and responses to individual questions (Annex 1) to facilitate better cross-study comparisons. Additional leadership from the World Health Organization or similar organizations on assessing essential knowledge among healthcare workers could further improve research in Rwanda and similar settings.

## Conclusion

Although our findings indicate that CHWs possessed lots of correct information about COVID-19 and reported good adherence to COVID-19 prevention practices, they also commonly held misconceptions that over-exaggerated the dangers of COVID-19. Gaps in knowledge, training, supplies, and access to information point to a need for additional investment in supervision and credible informational systems to support CHWs in Rwanda and in other similar settings with established CHW network.

### What is known about this topic

- *Community health workers (CHW) are increasingly incorporated into national outbreak preparedness planning;*
- *Early in the COVID-19 pandemic, the international community recognized that CHWs´ trusted position in the community, training and experience in disease prevention and management positioned them to effectively respond to COVID-19 pandemic;*
- *COVID-19 being unprecedented, research conducted in Rwanda and similar settings indicated that the knowledge and practice of health providers around COVID-19 substantially varied*.

### What this study adds

- *Rwandan CHWs possessed good level of correct knowledge about COVID-19 and reported good adherence to preventive practices; however, their COVID-19 knowledge was not associated with engaging in preventive activities;*
- *CHWs reported misconceptions around populations at risks, routes of transmission and the severity of COVID-19;*
- *Our findings point to broader gaps in the current CHWs system in Rwanda, including training, effective communication strategies and resources, which should be addressed to enhance preparedness for future outbreaks*.

## Annex

**Annex 1**: COVID-19 knowledge questions (In English and Kinyarwanda) that were used in this paper (MS Word 2010-37 K)

## References

[1] Bhutta Z, Lassi SZ, Pariyo WG, Huicho L (2010). Global experience of community health workers for delivery of health related millennium development goals: a systematic review, country case studies, and recommendations for scaling Up summary GLOBAL EXPERIENCE OF COMMUNITY HEALTH WORKERS FOR DELIVERY. World Heal Organ Glob Heal Work Alliance.

[2] Scott K, Beckham SW, Gross M, Pariyo G, Rao KD, Cometto G (2018). What do we know about community-based health worker programs? a systematic review of existing reviews on community health workers. Hum Resour Health.

[3] Boyce MR, Katz R (2019). Community health workers and pandemic preparedness: current and prospective roles. Front Public Heal.

[4] Anstey Watkins J, Griffiths F, Goudge J (2021). Community health workers´ efforts to build health system trust in marginalised communities: a qualitative study from South Africa. BMJ Open.

[5] Ballard M, Bancroft E, Nesbit J, Johnson A, Holeman I, Foth J (2020). Prioritising the role of community health workers in the COVID-19 response. BMJ Glob Heal.

[6] Bhaumik S, Moola S, Tyagi J, Nambiar D, Kakoti M (2020). Community health workers for pandemic response: a rapid evidence synthesis. BMJ Glob Heal.

[7] Chersich MF, Gray G, Fairlie L, Eichbaum Q, Mayhew S, Allwood B (2020). COVID-19 in Africa: care and protection for frontline healthcare workers. Global Health.

[8] Chowdhury P, Baidya S, Paul D, Debbarma P, Kalita B, Karmakar S (2021). Assessment of knowledge, attitude and practices among accredited social health activists (ASHAs) towards COVID-19: a descriptive cross-sectional study in Tripura, India. medRxiv.

[9] Feldman M, Lacey Krylova V, Farrow P, Donovan L, Zandamela E, Rebelo J (2021). Community health worker knowledge, attitudes and practices towards COVID-19: learnings from an online cross-sectional survey using a digital health platform, UpSCALE, in Mozambique. PLoS One.

[10] Gebremedhin T, Abebe H, Wondimu W, Gizaw AT (2021). COVID-19 prevention practices and associated factors among frontline community health workers in Jimma Zone, Southwest Ethiopia. J Multidiscip Healthc, Dove.

[11] Omoronyia O, Ekpenyong N, Ukweh I, Mpama E (2020). Knowledge and practice of COVID-19 prevention among community health workers in rural Cross River State, Nigeria: implications for disease control in Africa. Pan Afr Med J.

[12] Shrestha A, Thapa TB, Giri M, Kumar S, Dhobi S, Thapa H (2021). Knowledge and attitude on prevention of COVID-19 among community health workers in Nepal-a cross-sectional study. BMC Public Health.

[13] Rwanda Biomedical Center & Rwanda Ministry of health (2021 March 23).

[14] Nachega JB, Atteh R, Ihekweazu C, Sam-Agudu NA, Adejumo P, Nsanzimana S (2021). Contact tracing and the COVID-19 response in Africa: best practices, Key Challenges, and lessons learned from Nigeria, Rwanda, South Africa, and Uganda. Am J Trop Med Hyg.

[15] Niyigena A, Girukubonye I, Barnhart DA, Cubaka VK, Niyigena PC, Nshunguyabahizi M (2022). Rwanda´s community health workers at the front line: a mixed-method study on perceived needs and challenges for community-based healthcare delivery during COVID-19 pandemic. BMJ Open.

[16] Ndishimye P (2020). Knowledge, attitudes and preventive practices towards COVID-19 among frontline healthcare workers in Rwanda. Public Heal Bul.

[17] Schurer JM, Fowler K, Rafferty E, Masimbi O, Muhire J, Rozanski O (2020). Equity for health delivery: opportunity costs and benefits among Community Health Workers in Rwanda.

[18] Shapira G, Kalisa I (2016). Community performance-based financing impact evaluation 2013. Health Providers Follow-up Surve.

[19] Harris PA, Taylor R, Minor BL, Elliott V, Fernandez M, O´Neal L (2019). The REDCap consortium: building an international community of software platform partners. J Biomed Inform.

[20] Banda J, Dube AN, Brumfield S, Amoah AS, Reniers G, Crampin AC (2021). Knowledge, risk perceptions, and behaviors related to the COVID-19 pandemic in Malawi. Demogr Res.

[21] StataCorp (2017). Statistical Software: Release 15. StataCorp LLC.

[22] Karim N, Jing L, Austin Lee J, Kharel R, Lubetkin D, Clancy CM (2021). Lessons learned from Rwanda: innovative strategies for prevention and containment of COVID-19. Ann Glob Heal.

[23] Harper CA, Satchell LP, Fido D, Latzman RD (2021). Functional fear predicts public health compliance in the COVID-19 Pandemic. Int J Ment Health Addict.

[24] Neureiter A, Stubenvoll M, Kaskeleviciute R, Matthes J (2021). Trust in science, perceived media exaggeration About COVID-19, and social distancing behavior. Front Public Heal.

[25] World Health Organization (2020). An ad hoc WHO technical consultation managing the COVID-19 infodemic: call for action, 7-8 April 2020.

[26] Curtis V, Dreibelbis R, Sidibe M, Cardosi J, Sara J, Bonell C (2020). How to set up government-led national hygiene communication campaigns to combat COVID-19: a strategic blueprint. BMJ Glob Heal.

[27] Bertman V, Petracca F, Makunike-Chikwinya B, Jonga A, Dupwa B, Jenami N (2019). Health worker text messaging for blended learning, peer support, and mentoring in pediatric and adolescent HIV/AIDS care: a case study in Zimbabwe. Hum Resour Health.

[28] Willems A, Iyamuremye JD, Misage CN, Smith-Swintosky V, Kayiteshonga Y (2021). Co-creation and evaluation of nationwide remote training service for mental health education of community health workers in Rwanda. Front Public Heal.

[29] Datta R, Yadav AK, Singh A, Datta K, Bansal A (2020). The infodemics of COVID-19 amongst healthcare professionals in India. Med journal, Armed Forces India.

[30] Ho H-Y, Chen Y-L, Yen C-F (2020). Different impacts of COVID-19-related information sources on public worry: an online survey through social media. Internet Interv.

[31] Jemal B, Aweke Z, Mola S, Hailu S, Abiy S, Dendir G (2021). Knowledge, attitude, and practice of healthcare workers toward COVID-19 and its prevention in Ethiopia: a multicenter study. SAGE Open Med.

